# Older adults’ perceived capabilities for an active lifestyle in a rural german municipality: A cross-sectional study

**DOI:** 10.1371/journal.pone.0348937

**Published:** 2026-10-27

**Authors:** Sascha Vu-Eickmann, Alexandra Sauter, Zeineb Sassi, Patricia Vu-Eickmann, Dominik Morlok, Christine Bernardi, Lisa Boyer, Victoria Koschemann, Andrea Weber, Anne Herrmann, Thomas Okon

**Affiliations:** 1 Centre for Primary Care, University Hospital Regensburg and University of Regensburg, Regensburg, Germany; 2 Medical Sociology, Department for Epidemiology and Preventive Medicine, University of Regensburg, Regensburg, Germany; 3 Australian New Zealand Transplant and Cellular Therapies, Sydney, New South Wales, Australia; 4 Department of Orthopedics, University Hospital Center Regensburg, Bad Abbach, Germany; 5 Department of Epidemiology and Preventive Medicine, University of Regensburg, Regensburg, Germany; Dow University of Health Sciences, PAKISTAN

## Abstract

**Background:**

Physical activity (PA) is essential for healthy ageing, yet older adults in rural areas face particular constraints. This study applied Amartya Sen’s Capability Approach to examine how older adults in a rural German municipality perceive their opportunities for an active lifestyle and to identify factors associated with high PA engagement.

**Methods:**

A cross-sectional total population survey was conducted in a rural municipality in Bavaria, Germany. All residents aged ≥60 years (N = 593) were invited by post. Perceived capabilities were assessed using an instrument grounded in the Capability Approach. High PA engagement was defined as being physically active for at least 30 minutes on ≥4 days per week. Multivariable logistic regression was used to identify associated factors; multiple imputation by chained equations was used as a sensitivity analysis.

**Results:**

The response rate was 33.0% (N = 196); 166 respondents (84.7%) had complete data for the primary model. Mean age was 69.5 years (SD 7.6); 49.0% were men. Most participants (58.8%) rated their health as good, and 23.5% met the threshold for high PA engagement. Mean daily sedentary time was 6.12 hours (SD 3.37), higher in men than in women (6.64 vs 5.61 hours, p = 0.043). In the multivariable model, fewer perceived environmental opportunities were independently associated with lower odds of high PA engagement (OR 0.59 per SD, 95% CI 0.37–0.95), and poorer self-rated health showed a comparable association that did not reach conventional significance (OR 0.65 per SD, 95% CI 0.41–1.02). Under multiple imputation the association with poorer self-rated health was statistically significant (OR 0.60, 95% CI 0.38–0.94), as was male sex (OR 0.44, 95% CI 0.20–0.95), whereas the association with perceived environmental opportunities was no longer significant (OR 0.67, 95% CI 0.43–1.03). Age and social support were not associated with PA engagement. Living alone was associated with markedly lower perceived capabilities across several domains.

**Conclusions:**

Perceived environmental opportunities and health status, rather than information or social support, distinguished more from less active older adults in this rural setting. Capability-enhancing interventions should prioritise the built environment and should give particular attention to older adults living alone. Community-Based Participatory Research may help match provision to the capability sets of rural residents.

## Introduction

Global populations are ageing rapidly, resulting in a growing burden of late-life disease [1]. Promoting healthy ageing, the process of developing and maintaining functional ability to support well-being in older age, is therefore a pressing public health concern [2]. A physically active lifestyle is central to health in later life, with documented benefits including fall prevention, improved cardiorespiratory and metabolic health, enhanced musculoskeletal health, and increased life expectancy [3–5]. The World Health Organization (WHO) recommends that adults aged 65 years and above engage in at least 150 minutes of moderate-intensity aerobic physical activity or 75 minutes of vigorous-intensity activity weekly, or an equivalent combination [3].

Research from the United States, Canada, and Australia consistently shows that older adults in rural areas have more difficulty meeting these recommendations and exhibit higher obesity rates than their urban counterparts [6]. Limited access to PA programmes, poor infrastructure (e.g., absence of sidewalks or parks), and reduced social support all contribute to lower activity levels in rural populations [7]. This evidence base is Anglo-American, so transferability to the German context is rather limited as it differs substantially in healthcare systems, settlement structures, municipal provision and cultural norms around exercise differ substantially, thus findings do not transfer straightforwardly [8–10].

In Germany, data from the German Health Interview and Examination Survey for Adults (DEGS1) show that only 16.8% of women and 19.3% of men aged 60–69 years, and 11.0% of women and 16.5% of men aged 70–79 years, meet WHO physical activity guidelines [11]. These national estimates are not stratified by degree of urbanisation, so the extent to which rural older adults in Germany differ from their urban peers is not currently known from population data.

Strategies to promote physical activity in older populations range from individual-level approaches targeting lifestyle and motivation, such as social marketing campaigns [12], to community-based PA programmes [13]. Environmental approaches that modify contextual factors have shown promise for sustained behaviour change by making activity easier in everyday life [14]. For any of these interventions to work, they must be matched to the specific needs and preferences of the target group [15]. Understanding what shapes physical activity in rural older adults, and what might realistically improve it, is therefore a prerequisite for effective public health action.

A useful conceptual framework for examining such factors at both individual and environmental levels is Amartya Sen’s Capability Approach (CA) [16]. Developed originally in welfare economics, the CA defines capabilities as the perceived possibilities for action, shaped by personal attributes and environmental conditions that enable individuals to engage in health-promoting behaviours [17]. The framework has attracted growing interest in health promotion research, particularly in relation to physical activity [18–20]. Its analytic value here lies in the distinction between resources that are nominally available and those that individuals can actually convert into activity: a village may possess footpaths, courses and neighbours willing to help, yet these become capabilities only for those whose health, mobility and household circumstances allow them to be used. The factors governing that conversion, what Sen terms conversion factors, are precisely what a survey of perceived opportunities can capture and what provision statistics cannot [21,22]. Applying the CA to older adults’ physical activity in rural contexts may therefore identify constraints that are difficult to capture through conventional behavioural models [23,24].

Previous German research has identified barriers such as poor health, lack of company, and lack of interest, with significant gender and age differences [25]. The largest of these studies, a cross-sectional survey of primary care patients aged 70 and above, included rural participants but did not sample a rural population specifically, did not report results by degree of urbanisation, and was framed in terms of barriers rather than capabilities [25]; evidence from broader rural communities remains scarce. Subsequent reviews have highlighted the need for a deeper understanding of PA constraints, particularly through diverse theoretical approaches and in rural populations [26]. To our knowledge, no German study has applied the Capability Approach to physical activity in a rural population of older adults.

Given these gaps, this study aimed to describe how older adults in one rural German municipality perceive their capabilities for physical activity, and to identify the personal, social, and environmental factors associated with high PA engagement.

## Methods

### Data and sample

This cross-sectional study was conducted as part of an outcome evaluation of a five-year health promotion project targeting older adults residing in a rural municipality in southern Germany with 1,881 inhabitants. The area was classified as a Landgemeinde following the German Federal Institute for Research on Building, Urban Affairs and Spatial Development [27], meaning a community with fewer than 5,000 inhabitants. The study population comprised N = 593 residents aged 60 years and older, identified through the municipal registry and invited irrespective of health insurance status. Recruitment involved personalised invitation letters signed by the mayor, the local project manager, and the scientific lead; these letters included study information and participation details. To encourage response, a lottery incentive offering local vouchers was included. Data collection occurred in 2022 via an anonymous postal survey; questionnaires contained no identifying information (no IDs), and informed consent forms were returned separately in distinct envelopes to preserve participant anonymity. The study received ethical approval from the University of Regensburg Ethics Committee (approval no. 18-839-101).

Because the survey was anonymous by design and no register-based identifiers were retained, respondents cannot be compared with non-respondents on any characteristic. This is a deliberate consequence of the anonymity protocol and is addressed in the Limitations.

### Instrument

The paper-based German questionnaire comprised 56 items, of which 34 closed-ended questions were analysed here, grouped into “health and physical activity” (n = 22) and “sociodemographic data” (n = 12). Self-rated health was assessed by a single item asking participants to rate their general health on a five-point Likert scale, coded 1 = excellent to 5 = poor, so that higher scores denote poorer health. This coding is retained throughout the manuscript.

Physical activity (PA) was measured using the validated single-item PA measure developed by Milton et al. [28], which asks on how many days in a typical week the respondent is physically active for at least 30 minutes, with a six-point frequency scale from “never” to “more than five days per week”, a tool with strong reproducibility (r = 0.72–0.82).

Sedentary behaviour was assessed by a single item adapted from the World Health Organization’s Global Physical Activity Questionnaire, in which participants reported the average number of daily sedentary hours via open response [29]. This measure has demonstrated good test–retest reliability (Spearman’s rho = 0.78) and acceptable criterion validity against accelerometer data [30].

Capability items were grounded in Sen’s framework and operationalised using a set of questions developed by Sauter et al. from qualitative interviews with community-dwelling older adults [31]. The development and testing of this set of capability questions is described in detail elsewhere [32]. The instrument was adapted for this study population and validated through cognitive interviewing [31].

Two composite scores were formed from the capability items. The environmental opportunity score is the mean of four items on the opportunity to use nearby paths and places, to be in a safe environment, to move in a barrier-free environment, and to travel on foot or by bicycle (Likert scale; each 1 = fully agree to 5 = do not agree at all, so that higher scores denote fewer perceived opportunities). The social support score is the mean of five binary items on whether the respondent’s social environment supports their physical activity, whether family or friends exercise with them, help them find joint opportunities, motivate them, or relieve them of other duties to create time for activity. The questionnaire routes respondents who answer “no” to the first of these items past the remaining four; for these respondents the subsequent items were therefore coded as absent rather than missing. Respondents with fewer than three valid items were treated as missing.

Internal consistency for this sample is reported in Table A4 (see S1 Appendix) and discussed in the Limitations. Definitions and coding of all analysis variables are given in Table A1 (see S1 Appendix)

### Statistical analysis

This study was conducted and reported in accordance with STROBE guidelines for observational research [33]. Analyses were performed in R Studio 2026.07.1 (R version 4.6.1). Statistical significance was defined as two-sided p < 0.05. Tables with A can be found in the Appendix.

**Descriptive analysis.** Continuous variables are presented as means with standard deviations and as medians with interquartile ranges for skewed distributions. Categorical variables are reported as frequencies and valid percentages, that is, with missing responses excluded from the denominator; denominators are given in the tables.

Perceived capabilities measured on the five-point agreement scale are reported as the proportion of respondents selecting the highest response category (“fully agree”). This is a deliberately conservative operationalisation, chosen because the intermediate category (“tend to agree”) does not correspond unambiguously to a realisable opportunity. Proportions including the second category are also given in Table A5 to allow comparison with studies using a more inclusive threshold.

**Outcome definition.** The primary outcome was high PA engagement, defined as physical activity of at least 30 minutes on ≥4 days per week (the two highest categories of the six-point scale). This threshold is a study-specific definition of regular activity and is not equivalent to meeting WHO guidelines: four days of at least 30 minutes corresponds to at least 120 minutes per week, below the recommended 150 minutes, and the instrument records neither the actual duration beyond the 30-minute threshold nor the intensity of activity. The threshold was chosen because it separates respondents who are active on most days from those who are not, a distinction relevant for community-level provision, and because it corresponds to a natural cut point in the response scale. Results using the adjacent threshold are reported in the sensitivity analysis (Table A7).

**Missing data.** Missingness ranged from 0% (sex) to 10.7% (sedentary time) across the variables entering the primary model, and reached 60.7% for the project participation score, which was consequently not used as a covariate (Table A2). No fixed threshold was applied; this variable was excluded because more than half of its values were missing and it contributed nothing in preliminary models (all p > 0.6). Complete-case analysis was the primary approach, retaining 166 of 196 respondents (84.7%). As a sensitivity analysis, multiple imputation by chained equations was performed with 20 imputed datasets, ten iterations, and predictive mean matching (five donors); the imputation model included all analysis variables together with sedentary time, two further capability items, place of residence within the municipality, and disability status as auxiliary variables. Estimates were pooled using Rubin’s rules, and the fraction of missing information is reported for each coefficient.

**Primary analysis.** A multivariable logistic regression model was fitted with high PA engagement as the outcome and sex, age, self-rated health, the environmental opportunity score, and the social support score as covariates. Variables were selected a priori on theoretical grounds within the Capability Approach: sex and age as personal conversion factors, self-rated health as the individual resource most consistently associated with activity in older adults, and the two composite scores as environmental and social conversion factors respectively. All continuous predictors were z-standardised, so that odds ratios refer to a one-standard-deviation change; the standard deviation of age in this sample is 7.6 years. Results are presented as odds ratios with 95% confidence intervals.

Information accessibility was not included as a predictor of PA. Within the Capability Approach it is itself a capability rather than a conversion factor for activity, and it is analysed as an outcome in the capability-domain models (Table A6).

Interactions between sex and each of the two composite scores were tested and were not retained (p = 0.354 and p = 0.873). Only these two interactions were examined, because they had been specified a priori in the original submission; no further interaction terms were tested, given the number of events available.

**Capability-domain models.** Four secondary models examined which respondents reported each of four specific capabilities as present: information accessibility, use of nearby paths and places, participation in organised courses, and financial means for physical activity. Each was regressed on sex, age, and self-rated health. These models are exploratory and are reported in the (Table A6 in S1 Appendix).

**Model diagnostics.** Multicollinearity was assessed using variance inflation factors, all of which were below 1.3. Linearity of the logit for age was examined using the Box–Tidwell procedure (p = 0.231). Model fit was assessed using the likelihood-ratio test, the Hosmer–Lemeshow test (χ² = 4.56, df = 8, p = 0.803), and McFadden’s and Nagelkerke’s pseudo-R².

### Sample size and power

All eligible residents were invited (N = 593), so no sampling was performed. A simulation-based post-hoc power analysis (2,000 replications using the observed design and event rate) indicated 83% power to detect an odds ratio of 0.50 per standard deviation for self-rated health at N = 107, and correspondingly greater power at the achieved N = 166. Power for more moderate effects was limited: 59% for an odds ratio of 0.60 and 40% for 0.67. Null findings in this study should therefore not be read as evidence of absence.

### Multiple comparisons

The exploratory bivariate analyses reported in Tables A8a–A8c were corrected for multiple testing within each panel using the Benjamini–Hochberg procedure; uncorrected and corrected p-values are both reported.

## Results

### Participants

A total of 593 residents aged 60 years and older were identified and invited. Of these, 196 returned a completed questionnaire, a response rate of 33.0%. For the primary multivariable model, 166 respondents (84.7% of respondents) had complete data on the outcome and all covariates. Participant flow is shown in Fig 1.

**Fig 1:**
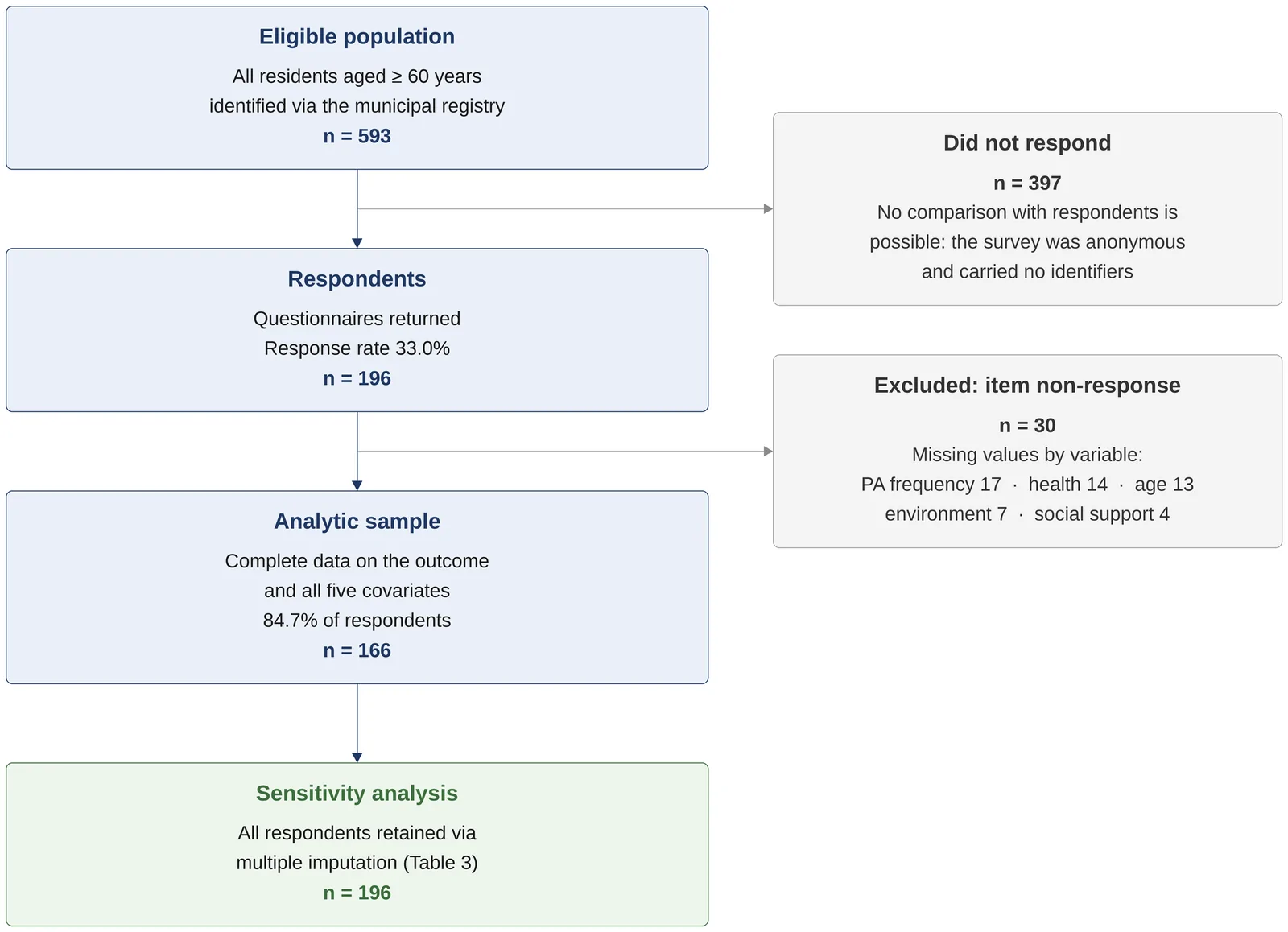
Participant flow.

Mean age was 69.5 years (SD 7.55; n = 183 with valid age), and 49.0% of respondents were men. Most were retired (69.5%); 24.3% lived alone and 75.7% lived with others. Of respondents with valid data on place of residence, 65.0% (119/183) lived within the main village and 35.0% (64/183) in the outlying settlements of the municipality. Self-rated health was reported as excellent or very good by 13.1%, good by 58.8%, and less good or poor by 28.0% (Table 1).

**Table 1:** Sociodemographic and health characteristics of the sample.

	male 49% (96)	female 51% (100)	total 100% (196)	Missing values
**Mean age (years)**	69.1 (SD 7.92) n = 90	69.9 (SD 7.20) n = 93	69.5 (SD 7.55) n = 183	13
**Age distribution**				13
*60-64*	40.0% (36/90)	23.7% (22/93)	31.7% (58/183)	
*65-74*	36.7% (33/90)	50.5% (47/93)	43.7% (80/183)	
*75-84*	18.9% (17/90)	21.5% (20/93)	20.2% (37/183)	
*85 and older*	4.4% (4/90)	4.3% (4/93)	4.4% (8/183)	
**Household situation**				15
*living alone*	18.0% (16/89)	30.4% (28/92)	24.3% (44/181)	
*living with others*	82.0% (73/89)	69.6% (64/92)	75.7% (137/181)	
**Marital situation**				16
*Married. living together*	77.3% (68/88)	60.9% (56/92)	68.9% (124/180)	
*Married. not living together*	2.3% (2/88)	1.1% (1/92)	1.7% (3/180)	
*Divorced*	4.5% (4/88)	3.3% (3/92)	3.9% (7/180)	
*Widowed*	12.5% (11/88)	30.4% (28/92)	21.7% (39/180)	
*Single*	3.4% (3/88)	4.3% (4/92)	3.9% (7/180)	
**Employment situation**				32
*Employed*	27.0% (24/89)	16.0% (12/75)	22.0% (36/164)	
*Retired*	67.4% (60/89)	72.0% (54/75)	69.5% (114/164)	
*Housewife/Houseman*	1.1% (1/89)	10.7% (8/75)	5.5% (9/164)	
*Unemployed*	1.1% (1/89)	0.0% (0/75)	0.6% (1/164)	
*Other*	3.4% (3/89)	1.3% (1/75)	2.4% (4/164)	
**Place of residence**				13
*Main village*	68.9% (62/90)	61.3% (57/93)	65.0% (119/183)	
*Outlying settlements*	31.1% (28/90)	38.7% (36/93)	35.0% (64/183)	
**Perceived health status**				14
*Excellent*	1.1% (1/89)	2.2% (2/93)	1.6% (3/182)	
*Very good*	16.9% (15/89)	6.5% (6/93)	11.5% (21/182)	
*Good*	60.7% (54/89)	57.0% (53/93)	58.8% (107/182)	
*Less good*	15.7% (14/89)	31.2% (29/93)	23.6% (43/182)	
*Poor*	5.6% (5/89)	3.2% (3/93)	4.4% (8/182)	
**Long-term care level (Pflegegrad)**				20
*Yes, any level (1–5)*	9.5% (8/84)	13.0% (12/92)	11.4% (20/176)	
*No*	90.5% (76/84)	87.0% (80/92)	88.6% (156/176)	
**Disabled person’s pass**				15
*Yes*	33.0% (29/88)	23.7% (22/93)	28.2% (51/181)	
*No*	67.0% (59/88)	76.3% (71/93)	71.8% (130/181)	
**PA Frequency Distribution**				17
*Never*	12.5% (11/88)	15.4% (14/91)	14.0% (25/179)	
*Not every week*	18.2% (16/88)	11.0% (10/91)	14.5% (26/179)	
*1 once per week*	12.5% (11/88)	9.9% (9/91)	11.2% (20/179)	
*2–3 x per week*	38.6% (34/88)	35.2% (32/91)	36.9% (66/179)	
*4–5 x per week*	13.6% (12/88)	20.9% (19/91)	17.3% (31/179)	
*>5 days per week*	4.5% (4/88)	7.7% (7/91)	6.1% (11/179)	
*Mean (SD)*	3.4 (SD 1.38) n = 88	3.6 (SD 1.51) n = 91	3.5 (SD 1.45) n = 179	
**High PA engagement (≥4 days/week)**	18.2% (16/88)	28.6% (26/91)	23.5% (42/179)	17
**Sedentary time (h/day), mean (SD)**	6.6 (SD 3.83) n = 87	5.6 (SD 2.77) n = 88	6.1 (SD 3.37) n = 175	21

Categorical variables are given as valid percentages with the underlying counts in parentheses, in the form % (n/ N valid responses); respondents who did not answer the item are excluded from N and are counted in the “Missing values” column. Denominators therefore differ between items. Continuous variables are given as mean (SD) with the number of valid responses beneath. Self-rated health is coded 1 = excellent to 5 = poor. “Long-term care level (Pflegegrad)” refers to the official German long-term care classification, assigned by the statutory medical review board on a five-point scale according to assessed need for assistance; “Yes” denotes any assigned level from 1 to 5. High PA engagement denotes physical activity of at least 30 minutes on ≥4 days per week (the two highest categories of the PA frequency item).

Respondents included in the primary model did not differ significantly from those excluded with respect to age, self-rated health, PA frequency, sedentary time, sex, or living alone (Table A3). They were, however, substantially less likely to live outside the main village (31.3% vs 70.6%, p = 0.001), which is relevant to the interpretation of the environmental findings and is addressed in the Limitations.

Percentages are valid percentages, i.e., calculated excluding missing responses. Self-rated health is coded 1 = excellent to 5 = poor.

### Physical activity and sedentary behaviour

Physical activity engagement varied widely. The median frequency of moderate-intensity PA of at least 30 minutes was 2–3 days per week. Among respondents with valid data (n = 179), 14.0% reported never being active, 36.9% were active on 2–3 days per week, and 23.5% met the study threshold for high PA engagement (≥4 days per week).

Mean daily sedentary time was 6.12 hours (SD 3.37; n = 175). Men reported more sedentary time than women (6.64 hours, SD 3.83, vs 5.61 hours, SD 2.77), a difference of approximately one hour per day (t-test p = 0.043). Given the skewed distribution of this variable, we also applied a rank-based test, which did not reach significance (Mann–Whitney p = 0.136); this difference should therefore be interpreted with caution. Sedentary time was not significantly associated with self-rated health (Spearman’s rho = 0.12, p = 0.108).

Respondents living in the outlying settlements reported substantially less sedentary time than those living in the main village (5.04 vs 6.70 hours per day, p = 0.001). They did not differ in PA frequency, in the proportion meeting the high PA threshold (OR 1.25, 95% CI 0.60–2.61), or in any capability item. Within this municipality, therefore, distance from the village centre was associated with less sitting but not with more structured activity.

### Perceived capabilities

Selected aspects of perceived capabilities relevant to physical activity were assessed across four domains and are shown in Figs 2 and 3. Percentages refer to respondents selecting “fully agree”; the corresponding proportions including “tend to agree” are given in Table A5.

**Fig 2:**
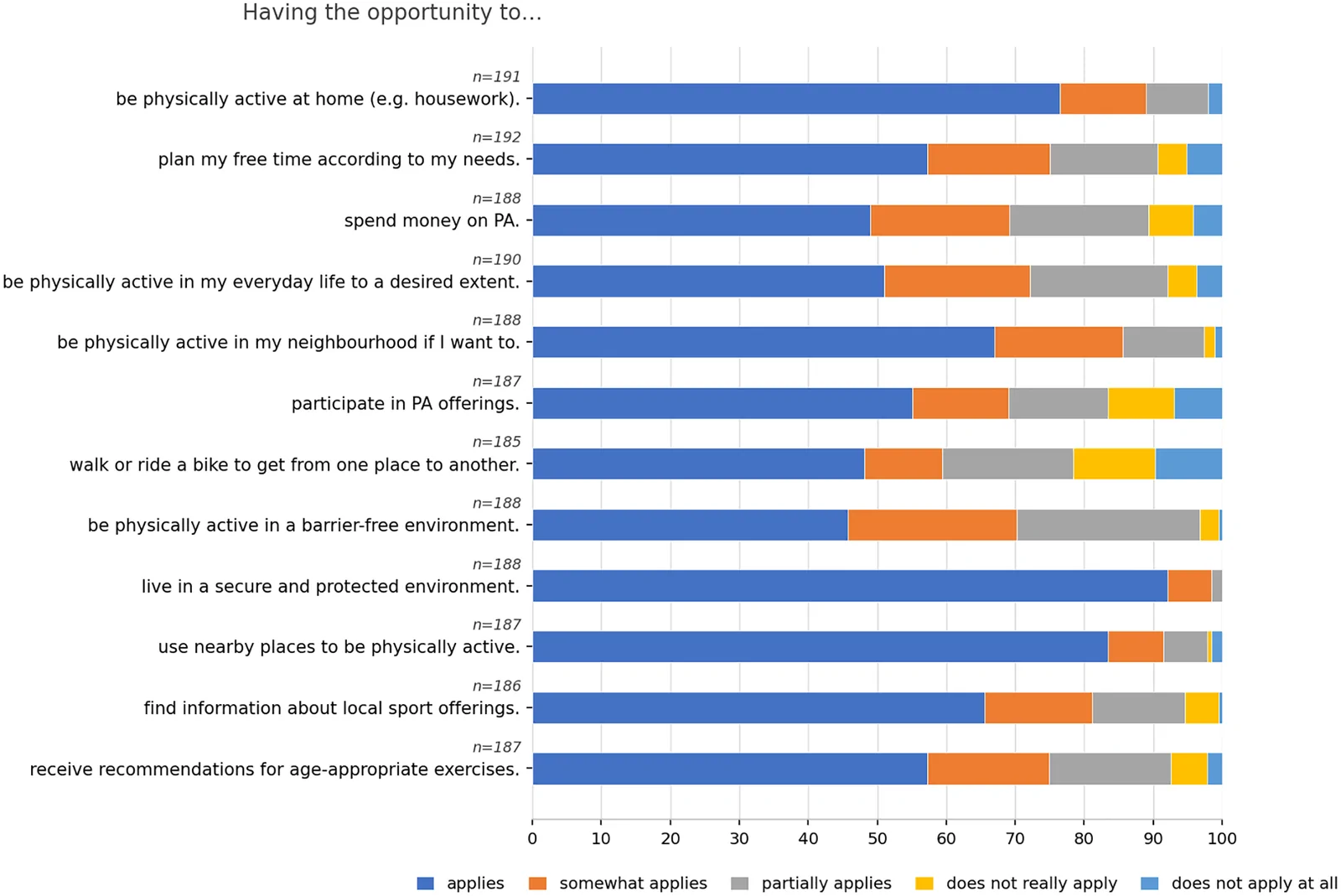
Perceived capabilities for an active lifestyle. Results are presented in %.

**Fig 3:**
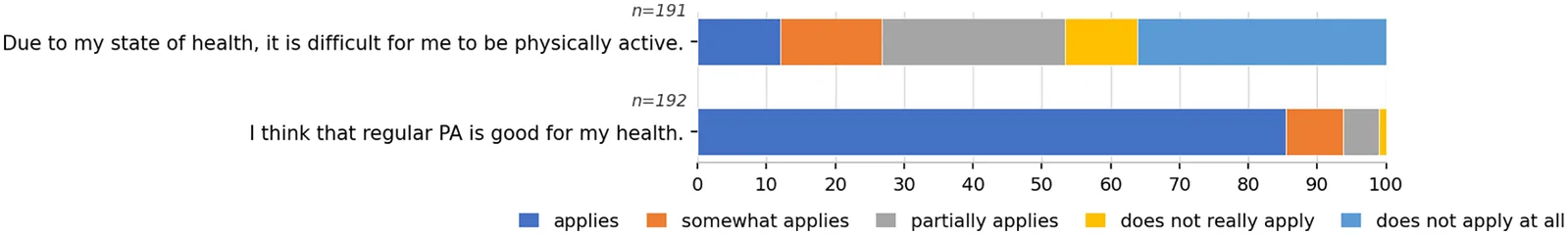
Relevance for an active lifestyle. Results are presented in %.

Knowledge and attitudes. A large majority (85.4%, n = 164) fully agreed that regular physical activity benefits health. Two thirds (65.6%, n = 122) knew where to find information about local PA offerings, and 57.2% (n = 107) knew where to find information about age-appropriate activity. Internal consistency of these two information items was good (α = 0.84). Only selected aspects of knowledge and attitudes were assessed; comprehensive health literacy was not measured.

**Living conditions.** More than half (57.3%, n = 110) felt able to organise their free time according to their own needs, and 48.9% (n = 92) felt able to make money available for physical activity. Respondents living alone reported markedly lower capabilities across several domains: compared with those living with others, they had lower odds of being able to use nearby paths and places (OR 0.24, 95% CI 0.10–0.57), to participate in organised courses (OR 0.33, 95% CI 0.16–0.70), to be as active in daily life as they wished (OR 0.44, 95% CI 0.21–0.90), and to receive social support for activity (OR 0.39, 95% CI 0.19–0.80); all remained significant after correction for multiple testing (Table A8b in S1 Appendix).

**Living environment.** Perceptions of the immediate residential environment were mixed. Almost all respondents fully agreed that they had the opportunity to be in a safe environment (92.0%, n = 173), and no respondent disagreed with this item. In contrast, only 45.7% (n = 86) fully agreed that they could move in a barrier-free environment, and only 48.1% (n = 89) that they could travel from place to place on foot or by bicycle. About half (51.1%, n = 97) felt able to be as physically active in daily life as they wished. Perceived safety was thus not a constraint in this community, whereas barrier-free access and everyday mobility were.

**Exercise environment.** Slightyl more than half (55.1%, n = 103) fully agreed that they had the opportunity to participate in organised courses. In the domain-specific models (Table A6), older age was associated with lower odds of reporting this capability (OR 0.51 per SD, 95% CI 0.35–0.74, p < 0.001), as was poorer self-rated health (OR 0.63 per SD, 95% CI 0.43–0.92, p = 0.017). Older age was likewise associated with lower odds of being able to use nearby paths and places (OR 0.63 per SD, 95% CI 0.41–0.97, p = 0.034), and poorer health with lower odds of having financial means available for activity (OR 0.68 per SD, 95% CI 0.48–0.97, p = 0.036). Information accessibility was not associated with sex, age, or health.

### Social support

Social support for physical activity was widely reported: 74.5% (n = 146) stated that their social environment supported them in being physically active, and among those, most also reported family or friends who exercised with them (79.1%), who helped them find suitable joint opportunities, who motivated them, or who relieved them of other duties. Internal consistency of the five-item scale was high (α = 0.92).

We found no evidence of a gender difference in social support. The proportion reporting support ranged from 70.7% to 85.3% in men and from 74.4% to 81.2% in women across the five items, and no item showed a significant difference before or after correction for multiple testing (Table A8a in S1 Appendix). Social support was positively correlated with PA frequency (Spearman’s rho = 0.23, p = 0.002) but was not independently associated with high PA engagement in the multivariable model.

### Multivariable analysis

Table 2 presents the primary model (N = 166; 40 respondents, 24.1%, met the high PA threshold). Fewer perceived environmental opportunities were independently associated with lower odds of high PA engagement (OR 0.59 per SD, 95% CI 0.37–0.95, p = 0.031); expressed in the opposite direction, each additional standard deviation of perceived environmental opportunity was associated with 1.69 times the odds of high engagement (95% CI 1.05–2.73). Poorer self-rated health showed an association of similar magnitude that did not reach conventional significance (OR 0.65 per SD, 95% CI 0.41–1.02, p = 0.059). Male sex was associated with lower odds of high engagement, also marginally (OR 0.46, 95% CI 0.21–1.00, p = 0.051). Age (OR 0.98, 95% CI 0.63–1.54) and social support (OR 1.14, 95% CI 0.77–1.69) were not associated with the outcome. The model was statistically significant overall (likelihood-ratio p = 0.006) and explained a modest share of variance (McFadden’s R² = 0.089; Nagelkerke’s R² = 0.140).

**Table 2:** Multivariable logistic regression for high physical activity engagement (≥4 days/week).

Variable	OR (95% CI)	p-value
Male sex (ref: female)	0.46 (0.21–1.00)	0.051†
Age (per 1 SD = 7.6 years)	0.98 (0.63–1.54)	0.947
Poorer self-rated health (per 1 SD)	0.65 (0.41–1.02)	0.059†
Fewer perceived environmental opportunities (per 1 SD)	0.59 (0.37–0.95)	0.031*
Social support for PA (per 1 SD)	1.14 (0.77–1.69)	0.521

N = 166 complete cases; 40 respondents (24.1%) met the threshold. Likelihood-ratio p = 0.006; McFadden’s R² = 0.089; Nagelkerke’s R² = 0.140. Legend: ** p < 0.01; * p < 0.05; ^†^ p < 0.10. Self-rated health is coded 1 = excellent to 5 = poor and the environmental score 1 = fully agree to 5 = do not agree at all, so for both an OR below 1 denotes lower odds of high PA engagement. OR = Odds Ratio; CI = Confidence Interval; PA = Physical Activity.

Under multiple imputation across all 196 respondents (Table 3), poorer self-rated health (OR 0.60, 95% CI 0.38–0.94, p = 0.026) and male sex (OR 0.44, 95% CI 0.20–0.95, p = 0.036) reached conventional significance, whereas the association with fewer perceived environmental opportunities was attenuated and no longer significant (OR 0.67, 95% CI 0.43–1.03, p = 0.068). The fraction of missing information was below 0.20 for all coefficients.

**Table 3:** Sensitivity analysis: the primary model under multiple imputation.

Variable	Complete case (N = 166) OR (95% CI)	p-value	Multiple imputation (N = 196) OR (95% CI)	p-value	FMI
Male sex (ref: female)	0.46 (0.21–1.00)	0.051†	0.44 (0.20–0.95)	0.036*	0.10
Age (per 1 SD = 7.6 years)	0.98 (0.63–1.54)	0.947	1.00 (0.65–1.54)	0.987	0.13
Poorer self-rated health (per 1 SD)	0.65 (0.41–1.02)	0.059†	0.60 (0.38–0.94)	0.026*	0.19
Fewer perceived environmental opportunities (per 1 SD)	0.59 (0.37–0.95)	0.031*	0.67 (0.43–1.03)	0.068†	0.06
Social support for PA (per 1 SD)	1.14 (0.77–1.69)	0.521	1.08 (0.73–1.59)	0.689	0.14

Twenty imputed datasets, ten iterations, predictive mean matching with five donors; estimates pooled using Rubin’s rules. FMI = fraction of missing information. ^*^ p < 0.05; ^†^ p < 0.10.

The association between self-rated health and PA engagement was robust across specifications (Table A7): the odds ratio ranged from 0.57 to 0.65 across six alternative model formulations, including the parsimonious model containing sex, age, and health only (OR 0.57, 95% CI 0.37–0.88, p = 0.010) and models using the adjacent outcome threshold. The association with the environmental opportunity score was somewhat less stable and was attenuated when the lower activity threshold was used.

## Discussion

### Health status, the built environment, and physical activity

In our sample, 58.8% of participants rated their health as good and 11.5% as very good, closely mirroring national data from the German Ageing Survey and DEGS1 [11,34,35], which supports the plausibility of our self-rated health estimates in this rural older population. Poorer self-rated health was associated with lower odds of high physical activity engagement, consistently across model specifications, and significantly so in the parsimonious model and under multiple imputation. Expressed in the direction more familiar from the literature, respondents whose self-rated health was one standard deviation better had roughly 1.5 to 1.8 times the odds of being active on four or more days per week.

The most consistent independent association in our data was with the perceived environment. Respondents who reported fewer opportunities to move in a barrier-free environment, to use nearby paths and places, and to travel on foot or by bicycle were markedly less likely to be regularly active, independent of health status, age, and sex. Within the Capability Approach this is the pattern one would predict: the municipality provides footpaths, courses and a health centre, but these constitute capabilities only for residents whose environment allows them to be reached and used [16,18,22]. The descriptive results help to locate the constraint. Perceived safety appeared not to be a barrier in this sample. 92% fully agreed that they could be in a safe environment and no respondent disagreed, whereas fewer than half fully agreed that their environment was barrier-free or that they could travel on foot or by bicycle. In this community, the constraint appears to lie less in perceived safety than in kerbs, gradients, distances and surfaces.

This distinction matters practically, because safety and accessibility imply different interventions. Lighting, traffic calming and neighbourhood watch schemes, as measures aimed at increasing safety, address a constraint these residents do not report. Continuous, even footpaths, dropped kerbs, benches at intervals, and accessible connections between outlying settlements and the village centre, as measures to improve environmental accessibility, address the constraint they do report [36–38].

### Living alone as a capability constraint

The most pronounced capability gradient we observed was associated with household composition. Respondents living alone were substantially less likely to report being able to use nearby paths and places, to participate in organised courses, to be as active as they wished in everyday life, and to receive social support for activity. These associations were large, were consistent in direction, and survived correction for multiple testing.

This is interpretable within the Capability Approach rather than as a simple motivational deficit. The same objective provision, a course in the health centre, a path around the village, converts into an actual opportunity at a different rate for someone with a partner who drives, notices, accompanies and reminds than for someone without. Household composition may thus be interpreted as a conversion factor. Respondents living alone may therefore represent an important group for capability-oriented interventions; they accounted for 24.3% of our sample. Measures that lower practical barriers may be particularly relevant in this group, for example transport arrangements, accompanied first visits, or buddy-based approaches; the latter improved exercise adherence in a randomised pilot study among older adults with functional limitations [39]. Distance to the exercise facility has likewise been identified as a negative predictor of adherence to prescribed exercise in older adults [40]. Evidence specific to rural German settings remains scarce.

### Age and the exercise environment

Older age was associated with lower odds of reporting the capability to participate in organised courses and to use nearby paths and places, but was not associated with high PA engagement itself once health and environment were accounted for. This suggests that the age gradient in our data runs primarily through the opportunity structure rather than through activity behaviour directly. Interventions that respond to this pattern (courses scheduled and located for people who no longer drive, provision within walking distance) are consistent with recommendations from the wider literature on age-tailored provision [26,41].

Information accessibility was not associated with age, sex, or health in our data. Two thirds of respondents knew where to find information about local offerings. Lack of knowledge and awareness has been reported as a potential barrier to physical activity in older adults [24]. In the present community, however, access to information was relatively widely reported, suggesting that informational access may not be among the most prominent perceived constraints.

### Sex differences

Men reported approximately one hour more sedentary time per day than women, a difference consistent with previous findings in this age group [42,43], although in our data it did not survive a rank-based test and should be regarded as suggestive. Men also had lower odds of high PA engagement, marginally in the complete-case model and significantly under multiple imputation.

We found no sex difference in social support for PA. Around three quarters of both men and women reported that their social environment supported their activity, and none of the five support items differed by sex. This is a null finding, and with 96 men and 100 women the study can exclude only moderate to large differences; it nevertheless suggests that in this community the lower activity levels observed in men are not explained by differential access to social support. Explanations resting on masculinity norms and help-seeking behaviour [44] remain plausible but are not testable with these data.

### Healthcare system and community interventions

General practitioners are well positioned to identify older adults who combine low self-rated health with limited PA-related capabilities [45,46]. Given the association between health status and engagement observed here, activity counselling and exercise referral, especially for those who are chronically ill, mobility-restricted, or housebound, represent an established and evidence-based component of general practice [47,48]. Community health managers could extend this reach by coordinating local trainers, running outreach programmes, and developing individualised plans [49–52]. General practitioners may also be aware which of their older patients live alone, which would allow the household dimension identified here to be considered in activity counselling. Our findings suggest that such outreach may be targeted by household composition and mobility rather than by information provision, and complements peer-support models in which motivated individuals help others maintain activity [53,54].

### Environmental conditions and infrastructure

Environmental factors set practical limits on what physical activity is realistically achievable. Essential built environment features (safe pedestrian and cycling routes, green spaces, adequate lighting, traffic-calming measures, continuous sidewalks, and accessible rest areas) collectively enable older adults to move freely and confidently [36–38,55]. Our data suggest that in this rural setting the accessibility components of this list matter more than the safety components. Outdoor fitness installations such as exercise trails and communal workout zones offer both physical and social benefits, though the evidence remains predominantly observational [55]. Randomised and quasi-experimental designs are needed to establish which environmental modifications most effectively support physical activity in rural populations [56,57].

### Future research directions

Our study, like much of the existing literature, is constrained in scope and design, and we make no claim to provide definitive answers. The alignment of our results with a broader body of evidence nevertheless indicates that the field should move beyond further descriptive work [9,13,14,31,58] toward studies that are pragmatic, grounded in community engagement, and directly aimed at narrowing capability gaps [13,59].

Community-Based Participatory Research. Our findings revealed considerable heterogeneity in perceived capabilities, suggesting that top-down strategies may lack the precision needed for rural contexts [15]. Co-developing interventions with local stakeholders could improve on the 33.0% response rate observed here and ensure that programmes are matched to the capability sets of rural residents [7,13,60].

Targeting conversion factors. The two most pronounced capability gradients in our data (household composition and the accessibility of the built environment) are both conversion factors rather than resources. Intervention research in rural settings might productively test whether addressing conversion directly (transport, accompaniment, path surfaces) outperforms increasing provision, which is the more common approach.

Economic models. Around half of our participants did not fully agree that they could make money available for physical activity, and this capability was associated with poorer health. Community-managed micro-grants or targeted subsidies for rural exercise programmes merit feasibility and cost-effectiveness research, with particular attention to reach among the most resource-limited [13,14,24,61].

Measurement. The self-reported nature of our data points to a need for methodological development in rural health research [10,62]. Ecological Momentary Assessment or wearable-based measurement could capture the dynamic, context-dependent nature of physical activity capabilities with higher ecological validity than surveys [9,10,63]. Validation studies are needed to assess feasibility, acceptability, and equity of such tools in rural populations [23].

Implementation and policy. Implementation science approaches, including natural experiments and pragmatic trials, can provide evidence on the real-world impact of policy changes such as infrastructure improvements or subsidised transport on physical activity capabilities in rural settings [9,23,24].

## Conclusion

Among older adults in this rural German municipality, the factors distinguishing the regularly active from the less active were the perceived accessibility of the built environment and, less consistently, self-rated health. Perceived safety, information access, and social support, all frequently invoked in this literature, were widely reported and did not differentiate. The sharpest capability gradient was associated with living alone. These findings point toward capability-enhancing interventions that target conversion rather than provision: barrier-free paths and connections between outlying settlements and village centres, and outreach designed around household circumstances and mobility rather than around information.

### Strengths and limitations

#### Strengths.

To our knowledge, this is the first study to assess community-dwelling older adults’ perceived capabilities for an active lifestyle in a rural German setting. We surveyed the total population of residents aged 60 and older in the municipality rather than a sample, which removes sampling error as a concern and gives complete coverage of the eligible population at the invitation stage. The assessment spans multiple capability domains within a single theoretical framework.

#### Limitations.

**Non-response.** The response rate was 33.0%, and because the survey was anonymous by design, respondents cannot be compared with non-respondents on any characteristic. The direction of any resulting bias is therefore unknown, although the over-representation of respondents aged 60–74 relative to the municipal age structure suggests that healthier and more mobile residents were more likely to participate. If so, capability estimates reported here are likely to be optimistic, and the true prevalence of capability constraints in this population is probably higher than our figures indicate.

**Attrition within the analytic sample.** Thirty of 196 respondents were excluded from the primary model owing to item non-response. Included and excluded respondents did not differ on age, health, activity, sedentary time, sex, or living alone, but those excluded were considerably more likely to live outside the main village (70.6% vs 31.3%). Since the environmental opportunity score is the most consistent predictor in our model and outlying residents are under-represented in the model, this association may be estimated with bias; the multiply imputed analysis, which retains all 196 respondents, produced a comparable estimate and provides partial reassurance.

**Measurement of physical activity.** Our outcome threshold of ≥4 days per week is a study-specific definition and does not correspond to WHO guideline attainment. The single-item instrument records the number of days on which respondents are active for at least 30 minutes; it does not record total duration, intensity, or the distribution of activity across the week. Our data therefore cannot speak to questions about weekly activity patterning, nor can they establish what proportion of this population meets WHO recommendations.

**Reliability of the environmental score.** Internal consistency of the four-item environmental opportunity score was low (α = 0.47). Inspection showed that one item, the opportunity to be in a safe environment, had almost no variance in this sample (92% fully agreed, no respondent disagreed) and a corrected item–total correlation of 0.08. The score is better understood as a formative index of distinct environmental constraints than as a reflective scale, and the coefficient associated with it should be read accordingly. Excluding the safety item changed the substantive result only marginally (Table A7). The social support scale (α = 0.92), the information accessibility items (α = 0.84), and the capability block as a whole (α = 0.72) showed acceptable to good consistency.

**Self-report and social desirability.** All data were self-reported, which may introduce recall bias, particularly for weekly activity and sedentary time. Participants may also have overstated activity levels or capability ratings to present themselves or their community favourably.

**Statistical power.** With 40 events in the primary model, power was adequate for strong associations but limited for moderate ones (59% for an odds ratio of 0.60). The non-significant findings for age and social support are therefore compatible with real but moderate associations, and should not be interpreted as evidence of no effect.

**Unmeasured socioeconomic position.** Analyses adjusted for sex and age but not for household income, educational attainment, or occupational history, all of which may confound the associations reported here.

**Scope of the capability assessment.** Health literacy was assessed only through selected items rather than comprehensively, which limits characterisation of information processing and utilisation capabilities. The project participation score could not be used as a covariate owing to 60.7% missingness.

**Design and generalisability.** The cross-sectional design precludes causal inference and cannot disentangle temporal relationships between health status, capability development, and activity. Definitions of rurality vary across the literature, complicating direct comparison even with studies asking similar questions. This is a single municipality of 1,881 inhabitants in eastern Bavaria; its demographic composition, topography, and level of infrastructure development may not be representative of rural Germany, and the environmental findings in particular are likely to be place-specific. Larger comparative studies across diverse rural communities would be needed to establish generalisability.

## Supporting information

S1 Appendix Tables A1-A9. (DOCX)

## Abbreviations

CA: Capability Approach
CI: confidence interval
FDR: false discovery rate
GP: general practitioner
MICE: multiple imputation by chained equations
PA: physical activity
SD: standard deviation
SES: socioeconomic status
WHO: World Health Organization
