# Supplementary material for: Older adults’ perceived capabilities for an active lifestyle in a rural german municipality: A cross-sectional study
Source: PLoS One. 2026 Oct 27;21(10):e0348937. doi: 10.1371/journal.pone.0348937 (PMC13712601; doi:10.1371/journal.pone.0348937)
Supplement: Supplementary file 1 [file pone.0348937.s001.docx]

# Appendix

## Table A1. Variable definitions and coding

| **Conceptual variable** | Item | Coding / operationalisation | Transformation |
| --- | --- | --- | --- |
| High PA engagement (outcome) | A02 (derived) | 0 = fewer than 4 days/week (scale points 1–4); 1 = ≥4 days/week (scale points 5–6) | Binary outcome |
| PA frequency | A02 | 1 = never; 2 = not every week; 3 = 1 day; 4 = 2–3 days; 5 = 4–5 days; 6 = more than 5 days per week | Dichotomised for the outcome |
| Self-rated health | A01 | 1 = excellent; 2 = very good; 3 = good; 4 = less good; 5 = poor (higher = worse) | z-standardised |
| Sedentary time | A03 | Hours per day sitting or lying, excluding sleep | Continuous |
| Environmental opportunity score | A06–A09 | Mean of four items, each 1 = fully agree to 5 = do not agree at all (higher = fewer opportunities) | z-standardised |
| Social support score | A18–A22 | Mean of five binary items, 1 = yes, 0 = no; A19–A22 coded 0 where the questionnaire skip applied | z-standardised |
| Information accessibility | A04 | 1 = fully agree vs 2–5 | Binary; outcome in Table A6 |
| Use of nearby paths and places | A06 | 1 = fully agree vs 2–5 | Binary; outcome in Table A6 |
| Course participation capability | A10 | 1–2 (agree or tend to agree) vs 3–5 | Binary; outcome in Table A6 |
| Financial capability | A15 | 1–2 (agree or tend to agree) vs 3–5 | Binary; outcome in Table A6 |
| Age | E01 | Years | z-standardised (SD = 7.6 years) |
| Sex | E06 | 1 = female; 2 = male | Binary: 0 = female, 1 = male |
| Place of residence | E07 | 1 = main village; 2 = outlying settlements | Binary |
| Living alone | E11a | 1 = yes; 2 = no | Binary |
| Disabled person’s pass | E04 | 1 = yes; 2 = no | Binary |

*Note: the binary derivations for items A04 and A06 use the highest response category only, whereas those for A10 and A15 combine the two highest categories. This follows the dichotomisation applied when the dataset was prepared and is reported here for transparency; the primary analysis does not depend on it.*

## Table A2. Missing data by analysis variable (N=196)

| **Variable** | n missing | % missing |
| --- | --- | --- |
| Sex | 0 | 0.0% |
| Social support score (A18–A22) | 4 | 2.0% |
| Environmental opportunity score (A06–A09) | 7 | 3.6% |
| Age | 13 | 6.6% |
| Self-rated health | 14 | 7.1% |
| PA frequency (outcome) | 17 | 8.7% |
| Sedentary time | 21 | 10.7% |
| Project participation score (not used as covariate) | 119 | 60.7% |
| Erroneous D-block composite (no longer used) | 71 | 36.2% |

*Complete cases for the primary model: 166 of 196 (84.7%).*

## Table A3. Respondents included in versus excluded from the primary model

| **Characteristic** | **Included**  **(n = 166)** | **Excluded**  **(n = 30)** | p-value |
| --- | --- | --- | --- |
| Age, years, mean (SD) | 69.3 (7.4) | 71.8 (8.9) | 0.268 |
| Self-rated health, mean (SD) | 3.19 (0.75) | 3.00 (0.82) | 0.376 |
| PA frequency, mean (SD) | 3.48 (1.47) | 3.38 (1.19) | 0.785 |
| Sedentary time, h/day, mean (SD) | 6.20 (3.42) | 5.35 (2.83) | 0.292 |
| Male sex, n (%) | 83 (50.0%) | 13 (43.3%) | 0.501 |
| Lives alone, n (%) | 38 (23.2%) | 6 (35.3%) | 0.267 |
| Lives outside the main village, n (%) | 52 (31.3%) | 12 (70.6%) | 0.001** |

*Welch’s t-test for continuous variables, chi-square test for categorical variables. ** p < 0.01.*

## Table A4. Internal consistency of the composite scales

| **Scale** | Items | n | Cronbach’s α |
| --- | --- | --- | --- |
| Social support for PA (A18–A22) | 5 | 185 | 0.92 |
| Information accessibility (A04, A05) | 2 | 186 | 0.84 |
| Capability item block (A04–A17) | 14 | 179 | 0.72 |
| Environmental opportunity score (A06–A09) | 4 | 183 | 0.47 |
| Environmental opportunity score excluding A07 | 3 | 183 | 0.52 |

*Item A07 (“opportunity to be in a safe environment”) showed almost no variance: 92.0% fully agreed, 6.4% tended to agree, 1.6% were undecided, and no respondent disagreed. Its corrected item–total correlation was 0.08.*

## Table A5. Perceived capabilities: proportion of respondents agreeing

| **Item** | **Fully agree**  **n (%)** | **Fully agree or tend to agree**  **n (%)** | Valid n |
| --- | --- | --- | --- |
| Knows where to find information on local PA offerings | 122 (65.6%) | 151 (81.2%) | 186 |
| Knows where to find information on age-appropriate PA | 107 (57.2%) | 140 (74.9%) | 187 |
| Has the opportunity to use nearby paths and places | 156 (83.4%) | 171 (91.4%) | 187 |
| Has the opportunity to be in a safe environment | 173 (92.0%) | 185 (98.4%) | 188 |
| Has the opportunity to move in a barrier-free environment | 86 (45.7%) | 132 (70.2%) | 188 |
| Has the opportunity to travel on foot or by bicycle | 89 (48.1%) | 110 (59.5%) | 185 |
| Has the opportunity to participate in organised courses | 103 (55.1%) | 129 (69.0%) | 187 |
| Can move around the neighbourhood as desired | 126 (67.0%) | 161 (85.6%) | 188 |
| Believes physical activity benefits health | 164 (85.4%) | 180 (93.8%) | 192 |
| Reports that health status impedes activity | 23 (12.0%) | 51 (26.7%) | 191 |
| Can be as physically active in daily life as desired | 97 (51.1%) | 137 (72.1%) | 190 |
| Has the opportunity to make money available for PA | 92 (48.9%) | 130 (69.1%) | 188 |
| Can organise leisure time according to own needs | 110 (57.3%) | 144 (75.0%) | 192 |
| Has the opportunity to be physically active at home | 146 (76.4%) | 170 (89.0%) | 191 |

*Each item was answered on a five-point scale from 1 = fully agree to 5 = do not agree at all. The main text reports the “fully agree” column.*

## Table A6. Capability-domain models: who reports each capability as present

| **Capability (outcome)** | Predictor | OR (95% CI) | p-value |
| --- | --- | --- | --- |
| **Information accessibility (A04)** | Male sex (ref: female) | 0.70 (0.37–1.35) | 0.289 |
|  | Age (per 1 SD) | 1.09 (0.78–1.53) | 0.616 |
|  | Poorer self-rated health (per 1 SD) | 0.85 (0.60–1.20) | 0.350 |
| **Use of nearby paths and places (A06)** | Male sex (ref: female) | 0.78 (0.33–1.84) | 0.562 |
|  | Age (per 1 SD) | 0.63 (0.41–0.97) | 0.034* |
|  | Poorer self-rated health (per 1 SD) | 0.68 (0.43–1.07) | 0.094† |
| **Participation in organised courses (A10)** | Male sex (ref: female) | 1.02 (0.50–2.08) | 0.956 |
|  | Age (per 1 SD) | 0.51 (0.35–0.74) | <0.001** |
|  | Poorer self-rated health (per 1 SD) | 0.63 (0.43–0.92) | 0.017* |
| **Financial means for PA (A15)** | Male sex (ref: female) | 0.86 (0.44–1.68) | 0.659 |
|  | Age (per 1 SD) | 1.02 (0.72–1.44) | 0.921 |
|  | Poorer self-rated health (per 1 SD) | 0.68 (0.48–0.97) | 0.036* |

*Information accessibility (A04): N = 169, McFadden’s R² = 0.009, LR p = 0.594; Use of nearby paths and places (A06): N = 169, McFadden’s R² = 0.075, LR p = 0.011; Participation in organised courses (A10): N = 170, McFadden’s R² = 0.125, LR p = <0.001; Financial means for PA (A15): N = 171, McFadden’s R² = 0.023, LR p = 0.177. These models are exploratory. ** p < 0.01; * p < 0.05; † p < 0.10.*

## Table A7. Sensitivity of the self-rated health association to model specification

| **Specification** | N | OR (95% CI) for poorer self-rated health | p-value |
| --- | --- | --- | --- |
| Primary model (complete case, ≥4 days/week) | 166 | 0.65 (0.41–1.02) | 0.059† |
| Omitting the social support score | 166 | 0.63 (0.40–0.99) | 0.045* |
| Parsimonious: sex, age, health only | 168 | 0.57 (0.37–0.88) | 0.010* |
| Environmental score excluding item A07 | 166 | 0.65 (0.41–1.02) | 0.062† |
| Lower outcome threshold (≥2–3 days/week) | 166 | 0.65 (0.44–0.98) | 0.037* |
| Multiple imputation (see Table 3) | 196 | 0.60 (0.38–0.94) | 0.026* |

** p < 0.05; ** p < 0.01; † p < 0.10.*

## Table A8. Exploratory bivariate associations

### Table A8a. Sex differences (male versus female as reference)

| **Outcome** | OR (95% CI) | p-value | FDR-adjusted p |
| --- | --- | --- | --- |
| Social support: environment supports my activity (A18) | 0.85 (0.45–1.62) | 0.621 | 0.745 |
| Social support: family or friends exercise with me (A19) | 1.22 (0.56–2.63) | 0.614 | 0.745 |
| Social support: finding joint opportunities (A20) | 0.83 (0.41–1.69) | 0.609 | 0.745 |
| Social support: being motivated (A21) | 1.66 (0.73–3.80) | 0.228 | 0.552 |
| Social support: being relieved of duties (A22) | 0.77 (0.35–1.69) | 0.519 | 0.745 |
| Knows where to find local PA information (A04) | 0.70 (0.38–1.29) | 0.256 | 0.552 |
| Can use nearby paths and places (A06) | 0.87 (0.40–1.88) | 0.719 | 0.785 |
| Barrier-free environment (A08) | 1.75 (0.98–3.12) | 0.059 | 0.552 |
| Mobile on foot or by bicycle (A09) | 1.38 (0.77–2.46) | 0.276 | 0.552 |
| Can participate in courses (A10) | 1.03 (0.58–1.83) | 0.924 | 0.924 |
| Can make money available for PA (A15) | 0.68 (0.38–1.21) | 0.189 | 0.552 |
| High PA engagement (≥4 days/week) | 0.56 (0.27–1.13) | 0.103 | 0.552 |

*Sedentary time, mean hours per day: men 6.64 (n = 87) versus women 5.61 (n = 88); Welch’s t-test p = 0.043, Mann–Whitney p = 0.136. * significant after Benjamini–Hochberg adjustment.*

### Table A8b. Living alone versus living with others

| **Outcome** | OR (95% CI) | p-value | FDR-adjusted p |
| --- | --- | --- | --- |
| Can use nearby paths and places (A06) | 0.24 (0.10–0.57) | 0.001 | 0.009* |
| Can participate in courses (A10) | 0.33 (0.16–0.70) | 0.004 | 0.013* |
| Can be as active in daily life as desired (A14) | 0.44 (0.21–0.90) | 0.024 | 0.042* |
| Can organise leisure time (A16) | 0.75 (0.37–1.50) | 0.411 | 0.480 |
| Knows where to find PA information (A04) | 0.82 (0.40–1.72) | 0.606 | 0.606 |
| Social support for PA (A18) | 0.39 (0.19–0.80) | 0.011 | 0.025* |
| High PA engagement (≥4 days/week) | 0.62 (0.25–1.53) | 0.298 | 0.417 |

*OR below 1 denotes lower odds among respondents living alone. * significant after Benjamini–Hochberg adjustment.*

### Table A8c. Spearman rank correlations

| **Variable pair** | ρ (Spearman) | n | p-value | FDR-adjusted p |
| --- | --- | --- | --- | --- |
| Self-rated health / PA frequency | -0.335 | 176 | <0.001 | <0.001* |
| Self-rated health / sedentary time | +0.123 | 172 | 0.108 | 0.155 |
| Self-rated health / environmental opportunity score | +0.322 | 180 | <0.001 | <0.001* |
| Age / PA frequency | -0.155 | 171 | 0.043 | 0.072 |
| Age / sedentary time | -0.020 | 168 | 0.799 | 0.799 |
| Age / social support | -0.093 | 180 | 0.213 | 0.267 |
| Environmental opportunity score / PA frequency | -0.230 | 177 | 0.002 | 0.005* |
| Social support / PA frequency | +0.233 | 176 | 0.002 | 0.005* |
| Social support / environmental opportunity score | -0.191 | 186 | 0.009 | 0.018* |
| Sedentary time / PA frequency | -0.022 | 172 | 0.778 | 0.799 |

*Self-rated health is coded 1 = excellent to 5 = poor and the environmental opportunity score 1 = fully agree to 5 = do not agree at all; for both, a higher value denotes a less favourable condition. p-values adjusted within the panel using the Benjamini–Hochberg procedure. * significant after adjustment.*

## Table A9. STROBE checklist

| Item No | Recommendation | Reported on page No |
| --- | --- | --- |
| **Title and abstract** | (a) Indicate the study's design with a commonly used term in the title or the abstract | 1 |
|  | (b) Provide in the abstract an informative and balanced summary of what was done and what was found | 1–2 |
| **Introduction** |  |  |
| Background/rationale | Explain the scientific background and rationale for the investigation being reported | 2–3 |
| Objectives | State specific objectives, including any prespecified hypotheses | 3 |
| **Methods** |  |  |
| Study design | Present key elements of study design early in the paper | 3 |
| Setting | Describe the setting, locations, and relevant dates, including periods of recruitment, exposure, follow-up, and data collection | 3 |
| Participants | (a) Give the eligibility criteria, and the sources and methods of selection of participants | 3–4 |
| Variables | Clearly define all outcomes, exposures, predictors, potential confounders, and effect modifiers. Give diagnostic criteria, if applicable | 4–5 |
| Data sources/ measurement | For each variable of interest, give sources of data and details of methods of assessment (measurement). Describe comparability of assessment methods if there is more than one group | 4 |
| Bias | Describe any efforts to address potential sources of bias | 4, 5, 7 |
| Study size | Explain how the study size was arrived at | 6 |
| Quantitative variables | Explain how quantitative variables were handled in the analyses. If applicable, describe which groupings were chosen and why | 5–6 |
| Statistical methods | (a) Describe all statistical methods, including those used to control for confounding | 5–6 |
|  | (b) Describe any methods used to examine subgroups and interactions | 6 |
|  | (c) Explain how missing data were addressed | 5 |
|  | (d) If applicable, describe analytical methods taking account of sampling strategy | 6 |
|  | (e) Describe any sensitivity analyses | 5, 12, 28 |
| **Results** |  |  |
| Participants | (a) Report numbers of individuals at each stage of study—eg numbers potentially eligible, examined for eligibility, confirmed eligible, included in the study, completing follow-up, and analysed | 6–7 |
|  | (b) Give reasons for non-participation at each stage | 7, 9 |
|  | (c) Consider use of a flow diagram | 9 |
| Descriptive data | (a) Give characteristics of study participants (eg demographic, clinical, social) and information on exposures and potential confounders | 7–8 |
|  | (b) Indicate number of participants with missing data for each variable of interest | 7–8, 26 |
| Outcome data | Report numbers of outcome events or summary measures | 10 |
| Main results | (a) Give unadjusted estimates and, if applicable, confounder-adjusted estimates and their precision (eg, 95% confidence interval). Make clear which confounders were adjusted for and why they were included | 11–12 |
|  | (b) Report category boundaries when continuous variables were categorized | 5, 26 |
|  | (c) If relevant, consider translating estimates of relative risk into absolute risk for a meaningful time period | n/a |
| Other analyses | Report other analyses done—eg analyses of subgroups and interactions, and sensitivity analyses | 10–11, 28–29 |
| **Discussion** |  |  |
| Key results | Summarize key results with reference to study objectives | 14, 17 |
| Limitations | Discuss limitations of the study, taking into account sources of potential bias or imprecision. Discuss both direction and magnitude of any potential bias | 17–18 |
| Interpretation | Give a cautious overall interpretation of results considering objectives, hypotheses, limitations, and similar studies | 14–17 |
| Generalisability | Discuss the generalisability (external validity) of the study results | 18 |
| **Other information** |  |  |
| Funding | Give the source of funding and the role of the funders for the present study and, if applicable, for the original study on which the p13resent article is based | n/a |

Page numbers refer to this version of the revised manuscript. Funding information is not stated in the manuscript, in line with PLOS ONE policy, and is declared in the submission form instead. Absolute risks are not reported because the analysis is based on odds ratios; event prevalences are given on page 10 and in Table 1.
